# Biosynthesis of conjugated linoleic acid: current status and future perspectives

**DOI:** 10.1186/s40643-025-00911-7

**Published:** 2025-07-11

**Authors:** Lu Lin, Mei-Li Sun, Kaifeng Wang, Jian Gao, Xiao-Jun Ji, Quanyu Zhao

**Affiliations:** 1 State Key Laboratory of Materials-Oriented Chemical Engineering, College of Biotechnology and Pharmaceutical Engineering, Nanjing Tech University, Nanjing, 211816 People’s Republic of China; 2 School of Pharmaceutical Sciences, Nanjing Tech University, Nanjing, 211816 People’s Republic of China; 3 School of Marine and Bioengineering, Yancheng Institute of Technology, Yancheng, 224051 People’s Republic of China

**Keywords:** Conjugated Linoleic acid, Linoleic acid isomerase, Biotransformation, De Novo biosynthesis, Synthetic biology

## Abstract

Conjugated linoleic acid (CLA) has received much attention for its beneficial physiological effects, particularly anticancer and metabolic control activity. However, traditional sources rely on extraction and chemical synthesis, both of which are not commercially viable. This article describes the research progress on sustainable alternative biosynthesis methods for the production of CLA, which is an attractive approach for commercial production because of its high stereoselectivity and convenient purification process. Here, we offer a comprehensive review of the current state and research progress in the biosynthesis of CLA, including production strains, biosynthesis mechanisms and improvements in the biosynthesis of CLA by genetic engineering. Finally, future research directions for improving the biosynthesis of CLA are defined in the light of recent advances, barriers and trends in this field.

**Supplementary Information:**

The online version contains supplementary material available at 10.1186/s40643-025-00911-7.

## Introduction

**Fig. 1:**
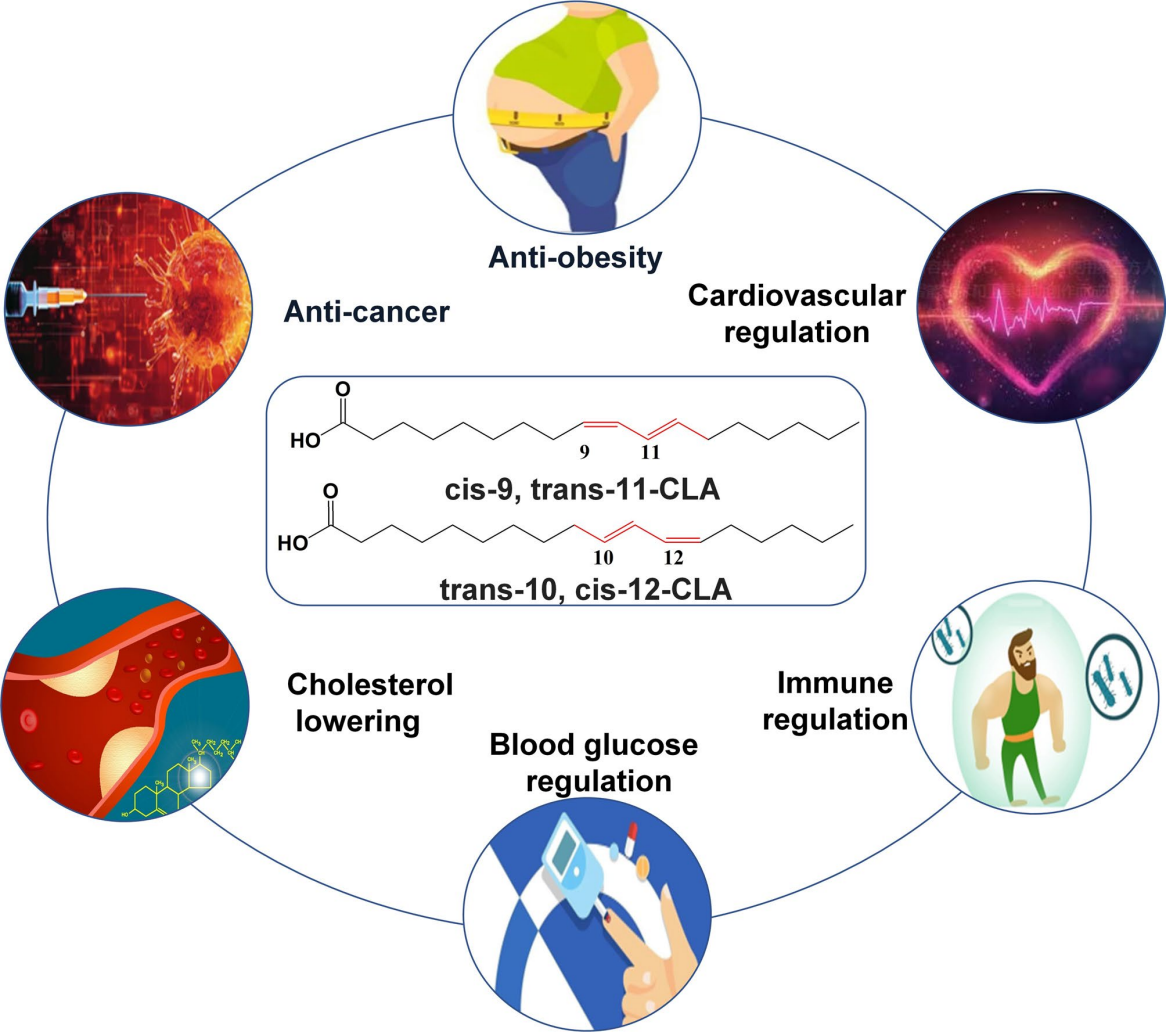
Applications of the two typical conjugated linoleic acid (CLA) isomers, cis-9,trans-11-CLA (c9,t11-CLA) and trans-10,cis-12-CLA (t10, c12-CLA)

Conjugated linoleic acid (CLA), which is found primarily in ruminant meat and milk, is a general name for the positional and geometric isomers of linoleic acid (LA) with a conjugated double bond. Among the many isomeric forms of CLA, cis-9,trans-11-CLA (c9,t11-CLA) and trans-10, cis-12-CLA (t10,c12-CLA) are the two most abundant and important isomers with notable physiological activity (Wu et al. 2024; Yang et al. 2017). Because of its unique molecular structure, CLA can alleviate the symptoms of diabetes, atherosclerosis and many other diseases, such as that t10,c12 CLA can reduce lipogenesis, and c9,t11-CLA has an antitumor effect, making it a health-promoting fatty acid (Fig. 1) (Chen et al. 2025; Dhar Dubey et al. 2019; George and Ghosh 2025; Gong et al. 2019; Sun et al. 2022).

The traditional sources of CLA are based on extraction and chemical synthesis, but due to the low content of CLA in animal fats, the steps needed to obtain the product with high purity are highly elaborate, making it difficult to apply the extraction process in industrial production. Currently, industrial CLA is primarily produced by alkaline isomerization of vegetable oils, but this method produces a mixture of several isomers, resulting in a very complicated downstream separation process. There is therefore an urgent need to search for novel and efficient CLA sources. Fortunately, it was found that certain microorganisms can biosynthesize CLA (Andrade et al. 2012; Liu et al. 2025), although the yield is generally low and is not sufficient to achieve economically viable industrial production. Nevertheless, obtaining CLA through biosynthesis is a promising alternative, as the process is not only mild but also characterized by a high degree of regio- and stereoselectivity (Wu et al. 2024). In this article, we will provide a comprehensive overview of the current state of CLA biosynthesis, including the commonly used microorganisms and related mechanisms, as well as strain engineering strategies for potential industrial CLA production. Finally, future perspectives of CLA biosynthesis are discussed.

## Microorganisms for the production of conjugated Linoleic acid

Table 1 summarizes the currently known microorganisms that can convert LA into CLA, most of which are anaerobic or facultative anaerobes that synthesize CLA when grown in media containing LA.

**Table 1:** Summary of the known bacteria that can convert Linoleic acid into conjugated Linoleic acid

Strains	Nos	Substrates (g/L)	Biocatalysts	Conjugated Linoleic Acid (CLA)				References
				Titer (g/L)	Conversion rate (%)	9, 11-CLA (%)	10, 12-CLA (%)	
*Butyrivibrio fibrisolvens*	A38	Linoleic acid (0.10)	Resting cells	0.08	80.0	95	5	Kim 2003
*Lactobacillus acidophilus*	L1	Linoleic acid (0.20)	Living cells	0.13	65.5	90	10	Alonso et al. 2003
	Q16	Linoleic acid (0.20)	Living cells	0.06	30.5	91	9	Alonso et al. 2003
	AKU1137	Linoleic acid (4.00)	Resting cells	1.50	37.5	100	0	Kishino et al. 2002
	AKU1137	Linoleic acid (5.00)	Living cells	4.90	98.0	100	0	Ogawa et al. 2001
*Lactobacillus casei*	E10	Linoleic acid (0.20)	Living cells	0.08	40.1	91	9	Alonso et al. 2003
	E5	Linoleic acid (0.20)	Living cells	0.11	55.5	88	12	Alonso et al. 2003
*Lactobacillus plantarum*	JCM1551	Linoleic acid (4.00)	Resting cells	2.02	50.5	100	0	Kishino et al. 2002
	JCM1551	Castor oil (5.00)	Living cells	2.70	54.0	100	0	Ando et al. 2004
	ZS2058	Linoleic acid (0.55)	Living cells	0.30	54.5	56	44	Yang et al. 2014
	PL 62	Linoleic acid (1.00)	Purified enzymes	0.65	65.0	47	53	Lee et al. 2007
	NCUL005	Linoleic acid (2.28)	Living cells	0.60	26.4	32	68	Zeng et al. 2009
*Lactobacillus reuteri*	ATCC55739	Linoleic acid (0.55)	Living cells	0.35	63.6	97	3	Yang et al. 2014
	ATCC55739	Linoleic acid (0.90)	Living cells	0.30	33.3	59	41	Lee et al. 2003b
*Lactobacillus breve*	IAM1082	Linoleic acid (4.00)	Resting cells	0.55	13.8	100	0	Kishino et al. 2002
*Bifidobacterium breve*	NCFB2257	Linoleic acid (0.55)	Living cells	0.23	42.0	99	1	Coakley et al. 2003
	NCFB2258	Linoleic acid (0.55)	Living cells	0.40	72.4	100	0	Coakley et al. 2003
	NCFB11815	Linoleic acid (0.55)	Living cells	0.22	39.1	99	1	Coakley et al. 2003
	NCFB8815	Linoleic acid (0.55)	Living cells	0.24	44.0	99	1	Coakley et al. 2003
	NCFB8807	Linoleic acid (0.55)	Living cells	0.13	23.3	99	1	Coakley et al. 2003
	LMC520	Linoleic acid (0.56)	Living cells	0.40	71.4	95	5	Coakley et al. 2003
*Bifidobacterium animalis*	Bb12	Linoleic acid (0.56)	Living cells	0.17	30.4	99	1	Yang et al. 2014
*Bifidobacterium lactis*	BB12	Linoleic acid (0.55)	Living cells	0.17	30.9	98	2	Coakley et al. 2003
*Enterococcus faecium*	M74	Soybean oil (10)	Living cells	0.73	7.30	100	0	Xu et al. 2006
*Megasphaera elsdenii*	YJ-4	Linoleic acid (0.02)	Living cells	0.01	50.0	15	85	Kim et al. 2002
*Pediococcus acidilactici*	AKU1059	Linoleic acid (4.00)	Resting cells	1.40	35.0	100	0	Kishino et al. 2002
*Propionibacterium freudennreichii*	P-6 Wiesby	Linoleic acid (0.75)	Living cells	0.27	35.3	93	7	Jiang et al. 1998
	9093	Linoleic acid (0.50)	Living cells	0.11	22.2	90	10	Jiang et al. 1998
	ATCC 6207	Linoleic acid (0.10)	Living cells	0.02	23.2	75	25	Jiang et al. 1998
*Propinibacterium freudenreichii subsp. shermanii*	56	Soybean oil (10)	Living cells	1.09	10.9	83	17	Xu et al. 2006
	51	Soybean oil (10)	Living cells	1.65	16.5	85	15	Xu et al. 2006
	23	Soybean oil (10)	Living cells	0.81	8.10	75	25	Xu et al. 2006
*Propionibacterium acnes*	No. 27	Linoleic acid (0.02)	Living cells	0.02	85.0	0	100	Verhulst et al. 1987

Where: 9, 11-CLA represents: c9, t11-CLA and t9, t11-CLA; 10, 12-CLA represents t10, c12-CLA

### Strains that primarily synthesize the c9,t11-CLA isomer

The naturally occurring microorganisms capable of synthesizing CLA include rumen bacteria, as well as species of the genera *Lactobacillus*, *Bifidobacterium*, *Propionibacterium*, and *Clostridium*, which mostly generate the c9,t11-CLA isomer as the main product. Rumen bacteria are the first group of strains reported to have the ability to biosynthesize CLA, which is an intermediate product in the bioconversion of LA to produce vaccenic acid or stearic acid. *Butyrivibrio fibrisolvens* A38 is the most efficient strain for CLA production among rumen bacteria, with a conversion rate of nearly 40%, 95% which is c9,t11-CLA (Hunter et al. 1976; Kepler and Tove 1967). Researchers also analyzed the factors affecting the CLA conversion rate (Kim et al. 2000), such as the substrate concentration, anaerobic environment and glycolysis inhibitors (Yang et al. 2017). In addition, the activity of CLA reductase can directly affect the accumulation of CLA, as for example the CLA reductase of *B. fibrisolvens* TH1 can convert CLA into vaccenic acid, which in turn reduces the accumulation of CLA (Kim 2003). Conversely, *B. fibrisolvens* MDT-5, which does not contain a CLA reductase, has a higher CLA content (Fukuda et al. 2006).

Another group of bacteria with the ability to synthesize CLA are lactobacilli, and they are widely used in CLA biosynthesis. Several *Lactobacillus* species have been shown to have the ability to transform LA into CLA rich in the c9,t11-CLA isomer. *Lactobacillus brevis* was the first species of the genus to be shown to convert LA into CLA. Notably, immobilized *L. brevis* produced 5.5 times more CLA than free resting cells, while *L. brevis* ATCC 55,739 was found to convert LA into CLA even in vivo in humans (Liu et al. 2025). *Lactobacillus plantarum* is another species with a high CLA production capacity. Kishino et al. (2002) showed that the CLA yield of *L. plantarum* AKU1009a could reach up to 85%. While *L. plantarum* JCM1551 can utilize ricinoleic acid as the substrate for the synthesis of CLA, with a final product titer as high as 2.7 mg/L (Ando et al. 2004). *L. plantarum* ZS2058 was able to convert 54.3% of LA into CLA (Yang et al. 2014). And it was found that the concentration of resting cells, substrate concentration, yeast extract in the medium, and glucose content can affect the efficiency of CLA conversion in *L. plantarum* (Khosravi et al. 2015). In addition to CLA, various *Lactobacillus* species convert LA into 10-hydroxy-cis12-octadecenoic acid (10-HOE), 10,13-dyhydroxyl-octadecanoic acid (10,13-diHOA), and 13-hydroxy-cis9-octadecenoic acid (13-HOE), with examples including *L. brevis* LTH2584 (Chen et al. 2016), *Lactobacillus rhamnosus* LGG (Yang et al. 2013), *Lactobacillus acidophilus* NCFM (Yang et al. 2013), L. *plantarum* AKU1009a (Kishino et al. 2002, 2011a, b, 2013), *L. plantarum* ATCC8014 (Ortega-Anaya & Hernández-Santoyo 2015), *L. plantarum* ZS2058 (Chen et al. 2016)d *acidophilus* AKU1137 (Ogawa et al. 2001). It has also been shown that CLA generated by resting cells of *L. acidophilus* is mainly accumulated intracellularly (Li et al. 2011). In addition, the oxygen in the fermentation system was found to only affects the ratio between isomers and not the total amount of CLA (Lin et al. 2005).

Species of *Bifidobacterium* constitute another group of efficient CLA producers. Coakley et al. (2003) first reported that *Bifidobacterium* sp. has the ability to convert LA into CLA with a preference for the c9,t11-CLA isomer, with *Bifidobacterium breve* exhibiting the highest ability of CLA synthesis. Another study analyzed the ability of 150 *Bifidobacterium* strains to produce CLA and found four strains with biotransformation rates of more than 80% with LA, all of which were *B. breve*, with *B. breve* LMC017CLA reaching a rate of up to 90.0% (Chung et al. 2008), and it could also produce CLA with monoglycerol linoleate as the substrate, with a conversion rate of 78.8%. Gorissen et al. (2010) examined the ability of 36 strains of *Bifidobacterium* sp. to synthesize CLA, and the results showed that the ability of *B. breve* to produce CLA was significantly higher than that of other *Bifidobacterium* species. In addition to *B. breve*,* B*. *longum* could was also able to produce CLA, and the ability to synthesize CLA differed significantly between strains, e.g. using LA as the substrate, the conversion rate of *B. longum* DPC6320 was 43.4%, while that of *B. longum* DPC6315 was only 11.0% (Barrett et al. 2007; Hennessy et al. 2012). In addition, it was noted that *Bifidobacterium dentium* can also produce CLA, whereby *B. dentium* NCFB2243 reached a conversion rate of 29.0% (Gorissen et al. 2010).

In addition, some species of *Propionibacterium* and *Clostridium* are also able to produce CLA. Verhulst et al. (1987) reported for the first time that *Propionibacterium* species can be used for CLA production, among which *P*. *freudenreichii* subsp. *freudenreichii*, *P*. *freudenreichii* subsp. *shermanii*, and *P*. *acidipropionici* were all able to convert LA into c9,t11-CLA. Among *Clostridium* species, it was found that CLA was an intermediate product in the biotransformation of LA by strains that produce vaccenic acid, and the generated CLA was dominated by the c9,t11-CLA isomer when using *C*. *bifermentans*, *C*. *sporogenes*, or *C*. *sordelli* as the while-cell catalyst (Verhulst et al. 1985). Peng et al. (2007) found that *C. sporogenes* ATCC22762 could convert the LA into CLA when the reaction time was 30 min, at this time, CLA was dominated by c9,t11-CLA, and prolongation of the reaction resulted in a decrease of this isomer, while the content of t9,t11-CLA as well as t10,c12-CLA rose, eventually resulting in comparable titers of all three isomers.

### Strains that primarily synthesize the t10,c12-CLA isomer

In contrast to c9,t11-CLA, fewer strains are able to synthetize t10,c12-CLA as the major isomer. Verhulst et al. (1987) showed that eight strains of *Propionibacterium acnes* (ATCC6919, ATCC6921, VPl 162, VPl 163, VPl 164, VPl 174, VPl 186, and VPl 199) can convert LA into CLA, whereby the main product was t10,c12-CLA. In addition to *P. acnes*, strains of *Megasphaera elsdenii* are also able to produce predominantly t10, c12-CLA, reaching a proportion of up to 85% (Kim et al. 2002). In addition, a few *Lactobacillus* strains can convert LA into CLA with t10,c12-CLA as the dominant isomer, such as *L. plantarum* PL 62 (Lee et al. 2007)d *plantarum* NCUL005 (Zeng et al. 2009).

## Mechanisms for microbial conjugated Linoleic acid production

### Monoenzyme catalysis system

**Fig. 2:**
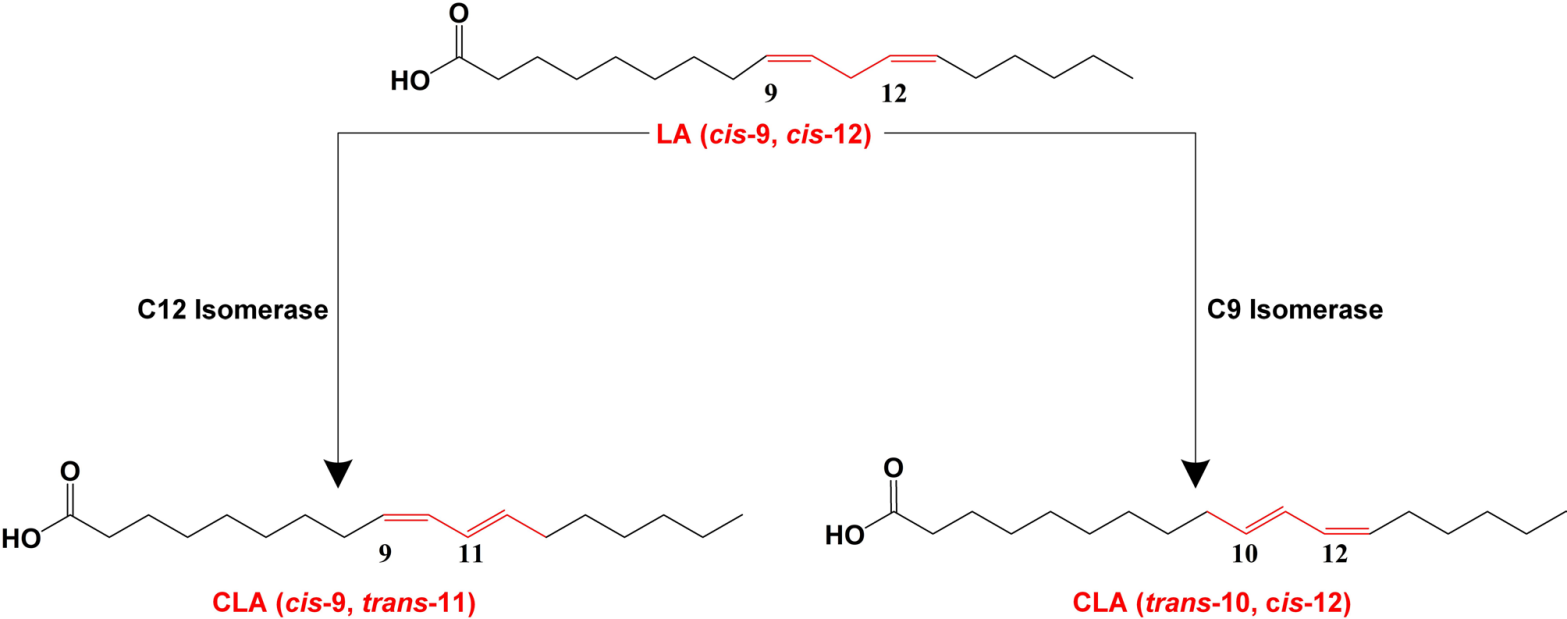
The conversion of LA into CLA catalyzed by two different LA isomerases. LA, linoleic acid, CLA, conjugated linoleic acid

The monoenzyme catalysis system is based on a single enzyme, i.e., LA isomerase (EC 5.2.1.5), which can produce two different CLA isomers. To date, three LA isomerases have been isolated and purified, which are derived from *Butyrivibrio fibrisolvens* (Kepler et al. 1966), *Lactobacillus reuteri* (Rosson et al. 2001), and *Propionibacterium acnes* (Deng et al. 2007). The first two LA isomerases modify the C12 double bond of the LA molecule to produce c9,t11-CLA (Fig. 2). They are membrane-bound proteins, unstable and highly susceptible to inactivation during purification, as well as substrate inhibition, making them unsuitable for the production of CLA by biotransformation. The LA isomerase derived from *P. acnes* acts on the C9 double bond of the LA molecule, with the product being t10,c12-CLA (Fig. 2). In contrast to the former two enzymes, it is an intracellular soluble protein, and is not sensitive to substrate inhibition. It is the only LA isomerase with a known crystal structure and the only one that has been successfully heterologously expressed in a variety of hosts including *Escherichia coli*, *Lactobacillus lactis*, *Saccharomyces cerevisiae*, *Yarrowia lipolytica*, etc., and even some plant cells such as tobacco and rice.

### Multienzyme catalysis system

**Fig. 3:**
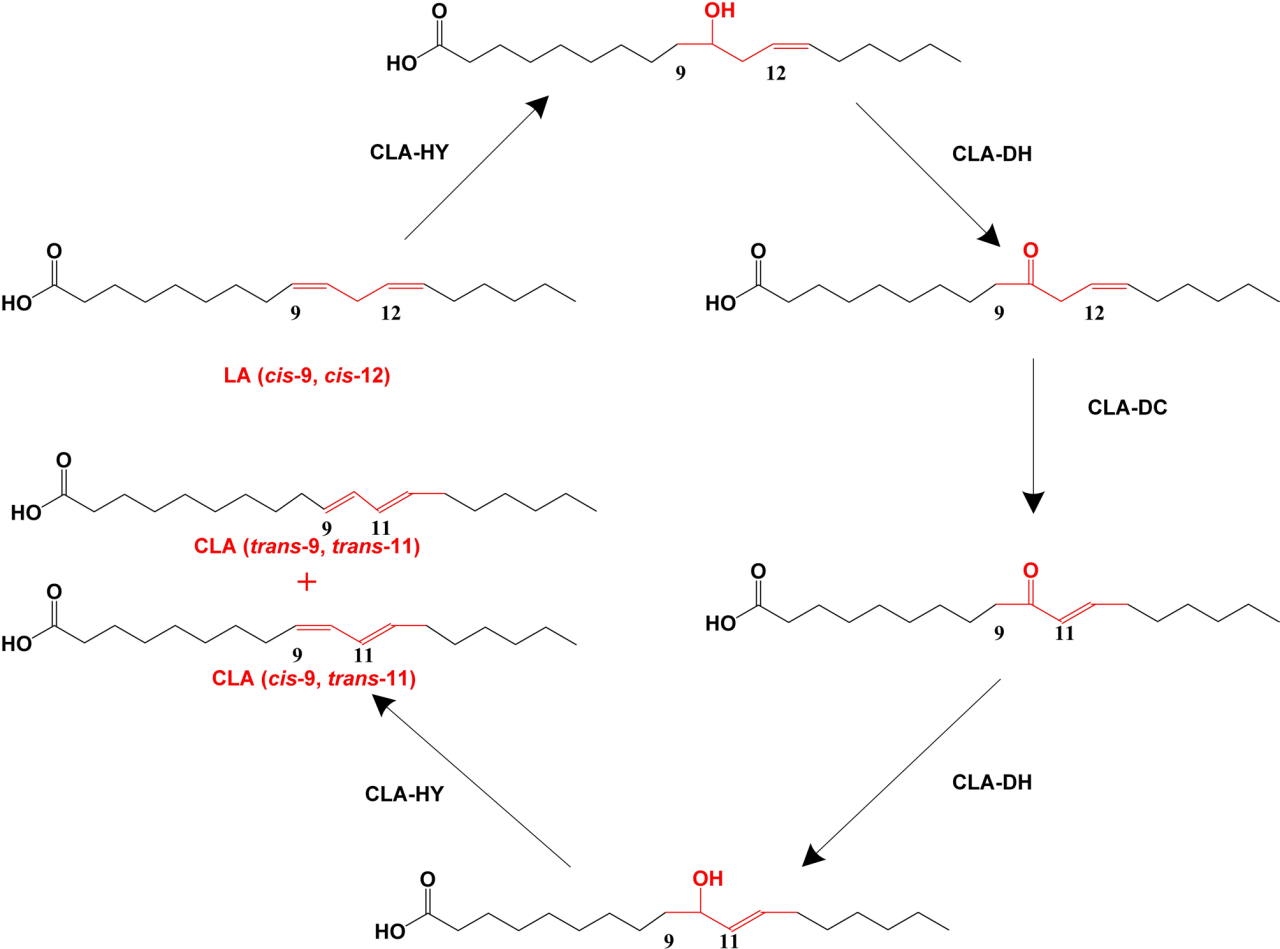
Proposed reaction scheme for the conversion of LA into CLA using the multienzyme catalysis system. LA, linoleic acid; CLA, conjugated linoleic acid; CLA-HY, CLA-DH, and CLA-DC are three enzymes constituting the LA isomerase system; CLA-HY, CLA oleate hydratase; CLA-DH, CLA short-chain dehydrogenase/oxidoreductase; and CLA-DC, CLA acetoacetate decarboxylase

The proposed multienzyme catalysis system involves multiple enzymes for the conversion of LA into CLA (Salsinha et al. 2018), whereby the isomerization of LA requires the production of intermediate hydroxy fatty acids, while the final product of the process is mostly c9,t11-CLA and to a lesser extent t9,t11-CLA (Ogawa et al. 2001). This mechanism is commonly observed during the in vitro production of CLA using *Lactobacillus* as a whole-cell catalyst. In 2011, a research team in Japan identified for the first time three enzymes that constitute a complex system for the conversion of LA into CLA in *L. plantarum* (Kishino et al. 2011a, b). Each of the three enzymes was expressed in *E. coli*, with the first enzyme (CLA-HY) present in the cytosolic fraction of the bacterium, and the other two enzymes (CLA-DH and CLA-DC) located in the soluble fraction. The CLA-DH shares sequence similarity with the short-chain oxidoreductase/dehydrogenase in the genome of *L. plantarum*, while CLA-DC is somewhat similar to acetoacetate decarboxylase. Only when the recombinant bacteria expressing the three enzymes were mixed, and with the addition of LA combined with cofactors (NADH, FAD and NADPH), the final products c9,t11-CLA and t9,t11-CLA could be detected. Thus, CLA could not be synthesized in the absence of any of these components, suggesting that the three enzymes are elements of a constitutive multienzyme system (Kishino et al. 2011a). Therefore, the multienzyme catalysis system for the conversion of LA into CLA was proposed based on the following 5 steps: (1) CLA-HY catalyzes the hydration of LA to produce the corresponding hydroxy fatty acid; (2) CLA-DH catalyzes the oxidation of the hydroxy fatty acid to produce a ketocarbonyl fatty acid; (3) CLA-DC catalyzes the isomerization of the double bond on the ketocarbonyl fatty acid molecule to produce a conjugated enone fatty acid; (4) CLA-DH catalyzes the inverse dehydrogenation of the conjugated keto fatty acid to produce a conjugated alkenohydroxy fatty acid; (5) CLA-HY catalyzes the inverse hydration of the conjugated alkenohydroxy fatty acid to produce c9,t11-CLA and t9,t11-CLA as the end products (Fig. 3).

## Strategies for conjugated Linoleic acid biosynthesis

### Biotransformation using natural strains

Currently, studies using natural microorganisms to convert LA into CLA generally using the following four methods: live cell fermentation, resting cell transformation, immobilized cell biosynthesis, and pure enzymatic biocatalysis, all of which have unique sets of advantages and disadvantages (Table 2).

**Table 2:** Methods for the production of conjugated Linoleic acid using natural strains

Methods	Details	Pros and cons	Typical examples
Living cell fermentation	Microbial growth and biosynthesis are coupled to each other, with growth and synthesis occurring at the same time	Easy to achieve high-density culture but cells cannot be reused	Lactobacillus lactis (Kim and Liu 2002) Bifidobacterium sp. (Oh et al. 2003)
Resting cell transformation	The resting microbes cannot grow due to the lack of certain essential nutrients, but the cells are still active due to the presence of various enzyme systems	Increased reaction activity but cumbersome steps	Propionibacterium fischeri (Rainio et al. 2001) Lactobacillus plantarum (Zhao et al. 2011)
Immobilized cell biosynthesis	Microbial cells with physiological functions are immobilised by certain methods and utilised as solid biocatalysts	Increased microbial cell reuse but reduced viability	Lactobacillus reuteri (Lee et al. 2003a) Lactobacillus delbrueckii and Lactobacillus acidophilus (Lin et al. 2005)
Pure enzymatic biocatalysis	Microbial cells are cultured at high density, and then they are fragmented and purified to obtain pure enzymes for biocatalysis	Higher catalytic efficiency but high cost of enzyme purification	Lactobacillus acidophilus and Propionibacterium freudenreichi (Lin et al. 2002) Lactobacillus delbrueckii (Lin 2006)

Kim and Liu (2002) identified 14 species of *Lactobacillus* in fermented milk with the ability to synthesize CLA from sunflower oil, among which *Lactobacillus lactis* I-01 had the highest synthesis ability and produced the maximum amount of CLA when the concentration of sunflower oil was 0.1 g/L. However, when the concentration of sunflower oil was greater than 0.2 g/L, the amount of CLA produced gradually decreased. Oh et al. (2003) studied the production of CLA by fermentation with *B. breve* and *B. pseudocatenulatum*, whereby the maximum amount of biomass was reached at 30 h of incubation, and the main isomer was c9,t11-CLA, which was distributed in the supernatant, with yields of 135–160 mg/L.

Rainio et al. (2001) cultured *Propionibacterium fischeri* in whey-supplemented medium, after which the cells were harvested, washed and placed into phosphate buffer containing 4.0 g/L LA for resting cell biotransformation. Notably, the conversion rate reached 46%, which was the highest yield reported at that time for the synthesis of CLA using resting cell biotransformation. Zhao et al. (2011) used the freeze-thaw method to permeabilize *L. plantarum* A6-1 F, which was then used to produce CLA. Using phosphate buffer (pH 7.0) as the biotransformation medium, a wet cell paste concentration of 150 g/L, LA concentration of 1.5 g/L, 37 °C, and a transformation time of 2 h, the highest yield of CLA reached 275.7 mg/L, which was nearly 20% higher than without permeabilization, and the main isomer was c9,t11-CLA.

Lee et al. (2003a) immobilized *Lactobacillus reuteri* ATCC 55,739 on silica gel, and the transformation reaction for 1 h at an LA concentration of 500 mg/L, pH 10.5, and 55 °C produced 175 mg/L of CLA, with a conversion rate of 35%. By contrast, free washed cells incubated for 1 h under the optimal reaction conditions produced only 32 mg/mL with a conversion rate of 6.4%. Thus, the CLA conversion rate of the immobilized cells was 5.5 times higher than that of the free cells. Lin et al. (2005) immobilized *Lactobacillus delbrueckii* ssp. *bulgaricus* CCRC 14,009 and *Lactobacillus acidophilus* CCRC 14,079 using both chitosan and polyacrylamide materials, respectively. At pH 7.0, the CLA yield was 121 µg for polyacrylamide-immobilized cells and 51.2 µg for chitosan, while the yield for free cells was only 29.4 µg.

Because most LA isomerases are difficult to isolate and purify, have poor thermal stability, and are easily inactivated during the extraction process, Lin et al. (2002) cultured *Lactobacillus acidophilus* in MRS broth, collected the cells by centrifugation, and then broke the wall with lysozyme followed by ultrasonication, and then subjected the crude enzyme to salting-out and dialysis, and finally concentrated the enzyme using a membrane with a molecular mass cutoff of 100 kD, which achieved a better purification effect. Lin (2006) compared the efficiency of *Lactobacillus* sp. washed cells and crude enzyme for the conversion of LA into CLA. The yield of CLA synthesized by washed cells was 209 mg/L, while that of the extracted crude enzyme was 8.5 mg/L, whereby the content of the individual isomers and the total CLA content was significantly lower than that of the transformation with washed cells, which might be related to the poor stability of free LA isomerase.

### Biotransformation or de novo biosynthesis using engineered strains

From the above analysis, it is clear that reliance on natural microbial cells does not allow for efficient biosynthesis of CLA. This is because the biocatalysts involved are inherently dependent on the properties of the cells, and natural microbial cells often do not fundamentally solve the problem of low catalytic efficiency due to insufficient enzyme amount and specificity. By contrast, pure enzymatic biocatalysis can solve the problem, because the products of this process are easy to separate, and the amount of enzyme can be adjusted according to the needs of the reaction, but it suffers from the high cost of enzyme purification, loss of enzyme activity during the reaction, and the complexity of operation.

For these reasons, researchers have genetically engineered more suitable host organisms to heterologously express LA isomerase from natural strains. Since the LA isomerase from *P. acnes* is able to synthesize 100% t10,c12-CLA isomer using LA as substrate (Deng et al. 2007), it has become a focus of research. Since *P. acnes* is a pathogenic microorganism that can cause diseases such as acne, it is not suitable for direct use in CLA synthesis. Therefore, researchers have attempted to heterologously express its LA isomerase-encoding gene, *Pa*LAI, in other safer species to efficiently and exclusively biosynthesize the t10,c12-CLA isomer (Table 3). Two studies from 2007 heterologously expressed the *Pa*LAI gene in *Escherichia coli* and *Lactobacillus lactis* (Deng et al. 2007; Rosberg-Cody et al. 2007), whereby the conversion of LA into CLA reached 50% and 40%, respectively. Moreover, the yield of t10,c12-CLA in *L. lactis* reached 0.2 g/L, and it reduced the viability of SW480 rectal cancer cells to 7.9%. Hornung et al. (2005) heterologously expressed *Pa*LAI in *Saccharomyces cerevisiae* and tobacco seedlings, demonstrating the substrate preference of *Pa*LAI. Since rice is a richer source of free fatty acids, Kohno-Murase et al. (2006) heterologously expressed *Pa*LAI in rice seedlings and found that the content of the product t10,c12-CLA reached 1.3% of the total rice lipids. Moreover, this genetically modified rice was used as feed in animal experiments to study the physiological effect of t10,c12-CLA, and also has the potential to be used as a nutraceutical product in the future.

**Table 3:** Engineering strategies for conjugated Linoleic acid biotransformation or de Novo biosynthesis using engineered strains

Chassis	Target genes	Methods	Substrates	Products	Productions	References
Tobacco	o*Pa*LAI	Heterologous expression	Linoleic acid	t10, c12-CLA	15.30% of total FAs	Hornung et al. 2005
Rice	o*Pa*LAI	Heterologous expression	–	t10, c12-CLA	1.30% of total FAs	Kohno-Murase et al. 2006
*Escherichia coli*	*Pa*LAI	Heterologous expression	Linoleic acid	t10, c12-CLA	0.08 g/L	Deng et al. 2007
*Lactococcus lactis*	*Pa*LAI	Heterologous expression	Linoleic acid	t10, c12-CLA	0.20 g/L	Rosberg-Cody et al. 2007
*Saccharomyces cerevisiae*	o*Pa*LAI	Heterologous expression	Linoleic acid	t10, c12-CLA	5.70% of total FAs	Hornung et al. 2005
*Trichosporon oleaginosus*	o*Pa*LAI	Heterologous expression	–	t10, c12-CLA	2.60% of total FAs	Görner et al. 2016
*Mortierella alpina*	o*Pa*LAI	Heterologous expression	–	t10, c12-CLA	1.20% of total FAs, 0.03 g/L	Hao et al. 2015
*Yarrowia lipolytica*	o*Pa*LAI	Heterologous expression	Glucose	t10, c12-CLA	5.90% of total FAs	Zhang et al. 2012
*Yarrowia lipolytica*	*Ma*FAD2 and o*Pa*LAI	Co-expression	Soybean oil	t10, c12-CLA	44.00% of total FAs, 4.00 g/L	Zhang et al. 2013
*Yarrowia lipolytica*	*Ma*FAD2 and o*Pa*LAI	Co-expression	Glucose	t10, c12-CLA	10.00% of total FAs	Zhang et al. 2013
*Yarrowia lipolytica*	o*Pa*LAI and ΔPEX10	Genetically modified	Safflower seed oil	t10, c12-CLA	7.40 g/L	Zhang et al. 2022
*Yarrowia lipolytica*	ΔPOX1–6, ΔDGA1, ΔDGA2, ΔARE1, ΔLRO1, *Yl*FAD2 and o*Pa*LAI	Genetically modified	Soybean oil	t10, c12-CLA	6.50% of total FAs, 0.302 g/L	Imatoukene et al. 2017
			Glucose		0.052 g/L	
*Yarrowia lipolytica*	*Ma*DGA1, *Ma*FAD2 and *Pa*LAI	Heterologous expression	Glycerol	t10, c12-CLA	0.130 g/L	Wang et al. 2019

total FAs, total fatty acids. Δ followed by letters refers to gene knockout. *Pa*LAI, linoleic acid isomerase gene from *P. acnes*; o*Pa*LAI, codon-optimized linoleic acid isomerase gene from *P. acnes*; *Ma*DGA1, diacylglycerol transferase 1 gene of *M. alpina*; *Ma*FAD2, Δ12 desaturase gene of *M. alpina*; PEX10, peroxisome biosynthesis factor 10 gene; POX1–6, peroxisomal acyl-CoA oxidase 1–6 genes; DGA1, diacylglycerol acyltransferase 1 gene; DGA2, diacylglycerol acyltransferase 2 gene; ARE1, Acyl-CoA cholesterol acyltransferase gene; LRO1, phospholipid: diacylglycerol acyltransferase gene; *Yl*FAD2, Δ12 desaturase gene of *Y. lipolytica*

To further achieve efficient biosynthesis of CLA, the researchers successively codon optimized *Pa*LAI and expressed it in the oleaginous yeast *Yarrowia lipolytica* using a strong promoter (Zhang et al. 2013), complemented by high expression of the FAD2 gene derived from *Mortierella alpina* which encodes the Δ12 desaturase catalyzing the conversion of oleic acid into LA. Using oleic acid-rich soybean oil as the substrate for whole-cell catalysis, they achieved a final yield of the target product, t10,c12-CLA of 4.0 g/L. Subsequently, they further improved the efficiency of biotransformation by cell permeabilization treatment to reduce product inhibition of the whole-cell catalyst (Zhang et al. 2016). After disrupting the β-oxidation pathway of *Y. lipolytica* to reduce the degradation of the substrate and the product by the whole-cell catalyst, when using safflower seed oil enriched in LA as the substrate, the yield of the target product t10,c12-CLA reached 7.4 g/L (Zhang et al. 2022). This successful case provides an effective strategy for high-level CLA production using *Y. lipolytica.*

However, although these methods can achieve high CLA yields, the presence of CLA in the form of free fatty acids can cause cytotoxicity and result in the termination of the reaction. Moreover, the biocatalysts are not recyclable, which increases the cost. Since *Y. lipolytica* has the ability to biosynthesize fatty acids de novo, studies also attempted to engineer it for the de novo biosynthesis of CLA from simple carbon sources (Zhang et al. 2012). In order to achieve this, researchers successively knocked out key genes of the β-oxidation pathway in *Y. lipolytica* (POX1-6) to block the degradation of fatty acids, overexpressed a codon-optimized *Pa*LAI gene, and achieved CLA biosynthesis from glucose as well as glycerol, but the yields were not high enough, reaching only 0.05 and 0.13 g/L, respectively (Imatoukene et al. 2017; Wang et al. 2019). This is because the studies did not manage to regulate the fatty acid fractions accumulated by *Y. lipolytica* to increase the proportion of LA, and the *Pa*LAI-encoded LA isomerase can only use free LA as substrate, whereas most of the fatty acids produced by *Y. lipolytica* accumulate in the form of triglycerides, resulting in the inaccessibility of the substrate for LA isomerase. This is another reason for the low yield of de novo CLA biosynthesis. In addition, the intermediate product free LA and the end product CLA are both cytotoxic to a certain extent, while the researchers did not try to enhance the fatty acid tolerance of the yeast chassis, illustrating the third reason for the low yield of de novo biosynthesis of the target product.

## Research needs and future directions

**Fig. 4:**
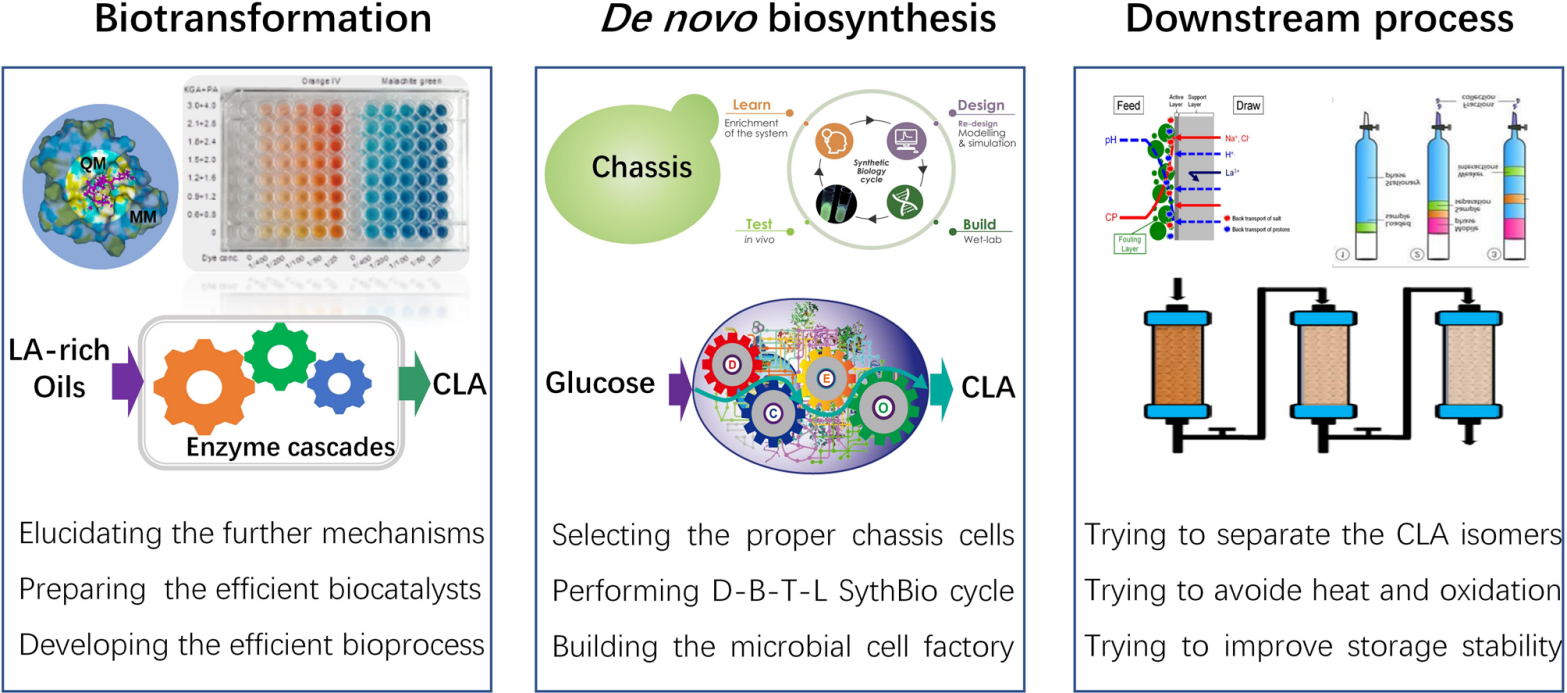
The proposed future research directions in the biosynthesis of CLA. LA, linoleic acid; CLA, conjugated linoleic acid

CLA has beneficial physiological activities, including antiobesity, anti-atherosclerotic, and even anticancer effects, making it a promising compound not only for functional foods, but also in clinical practice. Therefore, it is of great significance to strengthen the research on the synthesis of CLA. Currently, most CLA is produced by chemical isomerization, which requires harsh reaction conditions and results in a complex mixture of isomers, making it difficult to isolate a single isomer for applications in food and medicine. By contrast, biosynthesis of CLA has greater advantages. In this article, we have provided a comprehensive summary of the current status and research progress on sustainable alternative CLA biosynthesis methods, including potential production strains, biosynthesis mechanisms, and improving CLA biosynthesis by means of genetic engineering. Looking ahead, in order to further improve the efficiency and yield of CLA biosynthesis, making this process more economical and competitive, future research work can be carried out in the following aspects (Fig. 4).

First, the mechanism of CLA biosynthesis deserves further study, especially the differences between various bacterial strains, in order to lay a foundation for the establishment of more efficient biotransformation processes. On the basis of the mechanistic studies, the preparation and effective expression of biocatalysts, as well as the enhancement of the biotransformation process are important research aims. In addition, constructing microbial cell factories for the de novo biosynthesis of CLA from glucose is also an important goal. This requires strengthening synthetic biology research, including chassis cell selection, as well as upgrading the “design-build-test-learn” cycle to systematically and iteratively develop and optimize microbial cell factories for the de novo biosynthesis of CLA. Finally, one of the major challenges to the economics of CLA biosynthesis is the development of efficient and economical downstream processing technologies, which will accelerate its commercial application. Generally, there are two biosynthetic isomers of CLA, namely c9,t11-CLA and t10,c12-CLA. Although the product spectrum is much simpler than that of chemical synthesis methods, the two isomers may have unknown physiological effects when applied as a mixture. Therefore, developing efficient downstream processing techniques for CLA isomer separation is crucial for its pharmaceutical applications.

In conclusion, CLA, which has notable physiological activities including antiobesity and anticancer effects, is attracting increasing attention in the field of healthcare. With the design and/or discovery of novel production strains, the biosynthesis approach will offer significant opportunities to further decrease CLA production costs, which will greatly increase its application prospects.

## Electronic supplementary material

Below is the link to the electronic supplementary material.

Supplementary Material 1

## Data Availability

Not applicable.
