# Supplementary figures and images for: Biosynthesis of conjugated linoleic acid: current status and future perspectives
Source: Bioresour Bioprocess. 2025 Jul 11;12(1):72. doi: 10.1186/s40643-025-00911-7 (PMC12254105; doi:10.1186/s40643-025-00911-7)

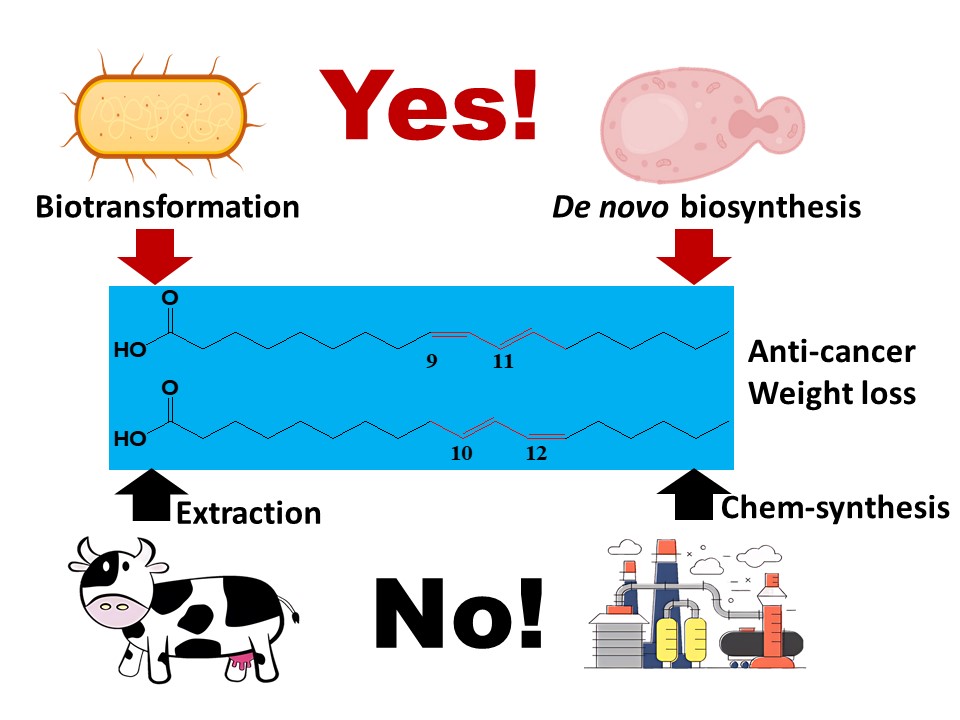

Supplement: Supplementary file 1 — Supplementary Material 1 [file 40643_2025_911_MOESM1_ESM.jpg]
